# IDH mutation, glioma immunogenicity, and therapeutic challenge of primary mismatch repair deficient IDH‐mutant astrocytoma PMMRDIA: a systematic review

**DOI:** 10.1002/1878-0261.13598

**Published:** 2024-02-09

**Authors:** Olfat Ahmad, Tahani Ahmad, Stefan M. Pfister

**Affiliations:** ^1^ Division of Pediatric Neurooncology Hopp Children's Cancer Center (KiTZ) Heidelberg Germany; ^2^ Division of Pediatric Neurooncology, German Cancer Research Center (DKFZ) German Cancer Consortium (DKTK) Heidelberg Germany; ^3^ Institute of Human Genetics University Hospital Heidelberg Heidelberg Germany; ^4^ University of Oxford Oxford UK; ^5^ King Hussein Cancer Center (KHCC) Amman Jordan; ^6^ Department of Pediatric Neuroradiology IWK Health Center Halifax Canada; ^7^ Dalhousie University Halifax Canada; ^8^ Department of Pediatric Hematology and Oncology Heidelberg University Hospital Heidelberg Germany

**Keywords:** IDH‐mutant glioma, immunogenicity, PMMRDIA

## Abstract

In 2021, Suwala *et al.* described Primary Mismatch Repair Deficient IDH‐mutant Astrocytoma (PMMRDIA) as a distinct group of gliomas. In unsupervised clustering, PMMRDIA forms distinct cluster, separate from other IDH‐mutant gliomas, including IDH‐mutant gliomas with secondary mismatch repair (MMR) deficiency. In the published cohort, three patients received treatment with an immune checkpoint blocker (ICB), yet none exhibited a response, which aligns with existing knowledge about the decreased immunogenicity of IDH‐mutant gliomas in comparison to IDH‐wildtype. In the case of PMMRDIA, the inherent resistance to the standard‐of‐care temozolomide caused by MMR deficiency is an additional challenge. It is known that a gain‐of‐function mutation of IDH1/2 genes produces the oncometabolite R‐2‐hydroxyglutarate (R‐2‐HG), which increases DNA and histone methylation contributing to the characteristic glioma‐associated CpG island methylator phenotype (G‐CIMP). While other factors could be involved in remodeling the tumor microenvironment (TME) of IDH‐mutant gliomas, this systematic review emphasizes the role of R‐2‐HG and the subsequent G‐CIMP in immune suppression. This highlights a potential actionable pathway to enhance the response of ICB, which might be relevant for addressing the unmet therapeutic challenge of PMMRDIA.

## Graphical abstract

Unmet therapeutic challenge of PMMRDIA. The co‐occurrence of an MMR deficiency with an *IDH* mutation poses a significant therapeutic challenge in PMMRDIA. Would addressing IDH mutation or subsequent DNA methylation, in conjunction with an ICB help overcome the resistance to ICB? ICB, immune checkpoint blocker; MMR, mismatch repair; PMMRDIA, primary mismatch repair deficient IDH‐mutant astrocytoma; R‐2‐HG, R‐2‐hydroxyglutarate; TMZ, temozolomide.

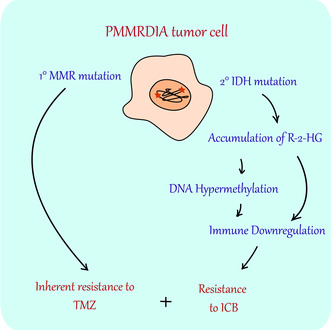

**Abbreviations**
5‐mC: 5‐methylcytosine
A: adenine
AML: acute myeloid leukemia
C: cytosine
CGGA: Chinese Glioma Genome Atlas
CMMRD: constitutional mismatch repair deficiency
CNS: central nervous system
CTA: cancer testis antigens
DCs: dendritic cells
DKFZ: German Cancer Research Centre
DNMT1: DNA methyltransferase 1
FACS: fluorescence‐activated cell sorting
FDG: 18F‐fluorodeoxyglucose
G: guanine
GBM: glioblastoma
G‐CIMP: glioma‐associated CpG island methylator phenotype
GO: gene ontology
HLA: human leukocyte antigen
HUGO: Human Genome Organization
ICB: immune checkpoint blocker
IHC: immunohistochemical
IRRDC: International Replication Repair Deficiency Consortium
LGG: low‐grade glioma
MAGE: melanoma‐associated antigens
MDS: myelodysplastic syndrome
MGMT: O‐6‐methylguanine‐DNA methyltransferase
MMR: mismatch repair
N7‐mG: N7‐methylated G
NFAT: nuclear factor of activated T cells
NKG2DL: NK group 2D ligand
O6‐mG: O6‐methylated G
PBMCs: peripheral blood mononuclear cells
PMMRDIA: Primary Mismatch Repair Deficient IDH‐mutant Astrocytoma
PMNs: polymorphonuclear leukocytes
PRISMA: Preferred Reporting Items for Systematic Reviews and Meta‐Analyses
PROSPERO: Prospective Register of Systematic Reviews
qPCR: quantitative PCR
R‐2‐HG: R‐2‐hydroxyglutarate
RNA‐seq: RNA‐sequencing
ROS: reactive oxygen species
RT: radiotherapy
T: thymine
TAMs: tumor‐associated microglia/macrophages
TCGA: The Cancer Genome Atlas
TCR: T cell receptor
TMB: tumor mutational burden
TME: tumor microenvironment
TMZ: temozolomide
t‐SNE: t‐distributed stochastic neighbor embedding
U: Uracil
WHO: World Health Organization

## 1 Introduction

### 1.1 Placement of *IDH* mutation in the glioma classification and placement of the PMMRDIA group within IDH‐mutant gliomas

After being first recognized in a single case of colorectal carcinoma in 2007 [1], *IDH* mutations have been identified in various types of human malignancies including – among others – acute myeloid leukemia (AML), intrahepatic cholangiocarcinoma, chondrosarcoma, and thyroid carcinoma [2, 3, 4, 5]. In glioma, the diagnostic and prognostic significance of IDH mutation was first identified in 2008 [6, 7]. Since 2016, the World Health Organization (WHO) has introduced molecular markers in its classification of central nervous system (CNS) tumors, allowing IDH‐mutant gliomas to be defined by their molecular features resulting in a more accurate diagnosis [8, 9]. In comparison to IDH‐wildtype gliomas of the same grade, IDH‐mutant gliomas are associated with a rather favorable prognosis [10], and they occur in ~ 80% of WHO grade II/III gliomas [11]. While only ~ 10% of glioblastoma (GBM) of WHO grade IV (current nomenclature is WHO grade IV diffuse glioma) are IDH‐mutant [12], they are particularly enriched in secondary diffuse glioma grade IV (73% vs. 3.7%), suggesting malignant transformation of a lower‐grade primary IDH‐mutant gliomas [11]. It is thought that more than half of the cases of recurrent IDH‐mutant gliomas develop secondary mismatch repair (MMR) deficiency, as a resistance mechanism to temozolomide (TMZ) [12], which is the most commonly used chemotherapeutic agent in treatment protocols of glioma (together with radiotherapy, following surgery) [13]. These recurrent IDH‐mutant gliomas with secondary MMR deficiency comprise the first of the two described entities of diffuse gliomas with co‐occurrence of *IDH* mutation, MMR deficiency, and hypermutation. The second being the recently defined Primary Mismatch Repair Deficient IDH‐mutant Astrocytoma (PMMRDIA) [14]. Both of these entities share an additional characteristic of TMZ resistance, which is inherent to primary mismatch repair deficient gliomas, hereafter referred to as dMMR gliomas [15]. In restricted t‐distributed stochastic neighbor embedding analyses conducted by Suwala *et al*. [14], primary and secondary MMR deficient IDH‐mutant astrocytomas were completely separated, hence, the definition of the distinct new group of PMMRDIA. Notably, in contrast to other IDH‐mutant gliomas, where O‐6‐methylguanine‐DNA methyltransferase (MGMT) promoter hypermethylation is a common feature in majority of the cases, PMMRDIA shows the highest frequency of unmethylated *MGMT* promotor [14]. Other than these two entities, the WHO classification of CNS tumors 2021 recognizes the following types of IDH‐mutant gliomas: diffuse astrocytoma (WHO grade II), anaplastic astrocytoma (WHO grade III), oligodendroglioma 1p/19q‐codeleted (WHO grade II), and anaplastic oligodendroglioma 1p/19q‐codeleted (WHO grade III) [9]. Needless to mention that similar to other types of astrocytoma, PMMRDIA is characterized by intact 1p/19q and high‐frequency *ATRX* inactivation [14].

### 1.2 MMR deficiency, MGMT promotor hypermethylation, and TMZ resistance

**Fig. 1:**
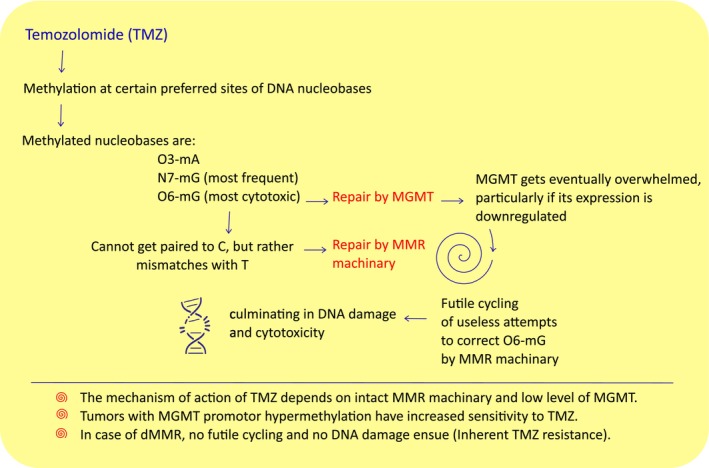
The mechanism of action of temozolomide. MGMT, O‐6‐methylguanine‐DNA methyltransferase; MMR, mismatch repair; N7‐mG, N7‐methylguanine; O3‐mA, O3‐methyladenine; O6‐mG, O6‐methylguanine. The figure illustrates the dependency of TMZ on MMR machinery, together with low levels of MGMT.

TMZ is an alkylating agent, that preferentially methylates DNA at O_3_ position of adenine (A), together with the N7 and O6 positions of guanine (G). Although N7‐methylated G (N7‐mG) is the major DNA adduct induced by TMZ, the cytotoxicity and mutagenicity are primarily attributed to the O6‐methylated G (O6‐mG) lesion [16]. O6‐mG lesions are directly repaired by MGMT [12]. Instead of pairing with cytosine (C), O6‐mG pairs with thymine (T) creating a mismatch that is recognized by DNA MMR machinery, which repairs the daughter strand but leaves behind the O6‐mG in the template strand for MGMT repair. A single MGMT molecule can only repair one alkyl adduct, and therefore, the repair of O6‐mG adducts is dependent on the number of MGMT molecules per cell and on the rate of MGMT regeneration [12]. The unrepaired O6‐mG leads to repeated attempts by the MMR pathway in a process called futile cycling, that results in replication‐associated DNA double‐strand breaks, which culminates in cell death [17]. Thus, TMZ cytotoxicity depends on an intact MMR pathway and low levels of MGMT, as is the case in MGMT promotor methylation (Fig. 1). This mechanism also explains the inherent resistance of dMMR tumors to TMZ [15]. Hence, Suwala *et al*. [14] did not show a difference in patients' outcomes for PMMRDIA with or without MGMT promotor methylation.

TMZ, similar to other alkylating agents, induces a mutagenic effect on the genome through DNA methylation and subsequent mismatches. In the model proposed by Choi *et al*. [12], TMZ resistance, which is marked by a hypermutator signature, occurs due to a TMZ‐induced mutation at a key amino acid of an MMR gene. Indeed, pre‐existing heterozygous deletions encompassing *MGMT*, or an MMR gene had been observed in some gliomas which later developed hypermutated recurrence, highlighting the survival advantage of these two events [12].

### 1.3 PMMRDIA: Diagnostic and therapeutic challenge

Since *IDH* enrichment has not been previously described in dMMR gliomas, the diagnosis of PMMRDIA could be overlooked as an IDH‐mutant glioma based on IDH immunohistochemical (IHC) staining alone, unless a suspicion of dMMR existed, especially in the context of treatment‐naïve glioma. Notably, the disparate global distribution of the autosomal‐recessive‐inherited constitutional mismatch repair deficiency (CMMRD) syndrome, which correlates with a large proportion of PMMRDIA cases, is concentrated in countries of high consanguinity such as the Middle East [18]. In cases of IDH‐mutant gliomas with intact 1p/19q or loss of ATRX as a surrogate in a child, adolescent, or young adult, a heightened level of suspicion is warranted, particularly if the histology exhibits high‐grade features. This recommendation is in accordance with Suwala *et al*. [14]. Not only in the typical setup of high CMMRD suspicion score, such as when there is a suggestive family history of Lynch syndrome spectrum tumors, or clinical features of neurofibromatosis 1 [19]. Indeed, newer versions of the molecular neuropathology methylation classifier developed by the German Cancer Research Centre (DKFZ), Heidelberg [20], will include PMMRDIA, which will aid the accurate diagnosis of this tumor group.

In the last decade, MMR deficiency has gained recognition as an indication for immune checkpoint blockers (ICBs). This marked a significant milestone with the first tissue‐agnostic cancer‐drug approval by the US Food and Drug Administration, following the publication of Le *et al*. 2015 [21]. Despite the fact that hypermutant gliomas are less responsive to ICB, due to their relative immunosuppressive microenvironment and the subclonal nature of the vast majority of mutations in MMR‐deficient tumors [22], several retrospective reports together with a recently published clinical trial by the International Replication Repair Deficiency Consortium (IRRDC) [23], have shown durable responses and prolonged survival of pediatric gliomas with high mutational burden and MMR deficiency [22]. Nevertheless, in the published cohort of Suwala *et al*. [14], none of the three patients who received ICB treatment during the course of the disease showed a notable response, attributed to *IDH* mutation or probably to small number of studied patients in the published cohort.

### 1.4 Understanding the molecular effects of an *IDH* event on a glioma

In humans, the IDH enzyme family includes three isoforms: IDH1, IDH2, and IDH3 [4]. All three forms are essential for several metabolic processes, such as the Krebs cycle, glutamine metabolism, lipogenesis, and redox regulation [24]. *IDH1/2* mutations that are associated with cancer tend to localize to the arginine (R) residue that is crucial for the recognition of isocitrate (R132 for *IDH1*, R140 or R172 for *IDH2*), with a vast majority of *IDH1* R132H events [7]. On the other hand, none of the three IDH3‐coding genes (*IDH3A*, *IDH3B*, and *IDH3G*) have been identified as significantly mutated genes in human cancers [4]. IDH enzymes normally convert isocitrate to α‐ketoglutarate (α‐KG) in the tricarboxylic acid (TCA) cycle. However, in IDH‐mutant gliomas, the mutated enzyme acquires a neomorphic function, converting α‐KG to R‐2‐hydroxyglutarate (R‐2‐HG) [25, 26, 27]. It is understood that R‐2‐HG interferes with the activity of multiple α‐KG‐dependent hydroxylases, exerting metabolic and epigenetic reprogramming of IDH‐mutant cancer cells, in addition to inducing paracrine effects on the TME [28].

#### 1.4.1 Metabolic effect of intracellular R‐2‐HG accumulation

The accumulation of R‐2‐HG disrupts various cellular metabolic pathways, contributing to tumorigenesis and malignant progression. One crucial aspect is the impact on cellular redox homeostasis. The conversion of α‐KG to R‐2‐HG consumes NADPH, a critical reducing agent involved in preventing oxidative stress. The reduced NADPH availability compromises the cell's ability to combat reactive oxygen species (ROS) and maintain redox balance, leading to increased oxidative stress and potential DNA damage, which further drives tumor development. Moreover, the alteration in the TCA cycle caused by *IDH* mutation has implications in energy metabolism. IDH‐mutant gliomas display decreased flux through the TCA cycle, leading to reduced ATP production via oxidative phosphorylation [29]. To compensate for the energy deficit, these tumors often rely on alternative metabolic pathways, such as aerobic glycolysis (the Warburg effect), to generate ATP and support their rapid proliferation. This shift in energy metabolism contributes to the characteristic metabolic phenotype observed in gliomas and is associated with increased glucose uptake, commonly observed in positron emission tomography imaging using 18F‐fluorodeoxyglucose (FDG) [30]. Furthermore, IDH‐mutant gliomas exhibit altered lipid metabolism. Studies have shown that *IDH* mutations can influence the expression and activity of lipid biosynthetic enzymes, leading to increased lipogenesis and lipid accumulation in tumor cells. Lipids serve as a crucial source of energy and building blocks for cell membranes, and dysregulated lipid metabolism in IDH‐mutant gliomas affects their growth and survival. Altogether, these metabolic changes create a unique metabolic profile in IDH‐mutant gliomas, making them distinct from other glioma subtypes [31]. This lays the groundwork for the development and utilization of several targeted IDH1/2 inhibitors in the treatment of IDH‐mutant glioma and other IDH‐mutant tumors [32].

#### 1.4.2 IDH‐induced epigenetic reprogramming of tumor cells

Epigenetic dysregulation exerted by an *IDH* event is a fundamental characteristic of gliomagenesis, in addition to the described metabolic effects. Among enzymes inhibited by R‐2‐HG, are demethylases, namely, TET family of 5‐methylcytosine hydroxylases [33] and lysine demethylases [34], leading to widespread alterations in DNA and histone methylation patterns, respectively. The aberrant DNA hypermethylation observed in IDH‐mutant gliomas is particularly evident at CpG islands, repressing critical tumor suppressor genes involved in cell cycle regulation, DNA repair, and apoptosis. This epigenetic silencing contributes to uncontrolled cell proliferation and tumor growth, which is the hallmark of IDH‐mutant gliomas, alternatively called as the glioma‐associated CpG island methylator phenotype (G‐CIMP) [4, 35]. Additionally, IDH mutations impact histone methylation, which alters chromatin structure and gene regulation, affecting chromatin accessibility and transcriptional activity. These changes can enhance the expression of oncogenes or suppress the tumor suppressor genes, thereby, driving glioma development. Furthermore, the epigenetic effect of *IDH* mutation may influence cellular differentiation processes. Additionally, R‐2‐HG can interfere with enzymes involved in cellular differentiation pathways, promoting a dedifferentiated state in glioma cells. This loss of normal cellular identity may contribute to the glioma's malignant potential and aggressive behavior [36]. The effectiveness of inhibiting methylation in IDH‐mutated glioma cells was supported by Turcan *et al*. [37], which demonstrated that decitabine, an inhibitor of DNA methyltransferase 1 (DNMT1) suppresses the proliferation of IDH‐mutant glioma cells both *in vitro* and *in vivo*. Likewise, Borodovsky *et al*. [38] observed that 5‐azacytidine, a cytidine analog that interferes with the activity of DNA methyltransferase, led to the regression of a patient‐derived IDH‐mutant glioma xenograft.

#### 1.4.3 Paracrine effect of 2‐HG on the TME

Elevated levels of R‐2‐HG have been detected both inside tumor cells and in the serum of several types of IDH‐mutant tumors [25, 26, 27]. This finding helps to elucidate how the accumulation of R‐2‐HG may lead to a survival advantage for glioma cells, despite potentially disrupting multiple metabolic pathways that compromise cellular fitness. It became evident that the export of excess intracellular R‐2‐HG into the tumor microenvironment (TME) and bodily fluids serves as a protective mechanism in tumor cells [28]. Subsequent import of R‐2‐HG to T lymphocytes in a paracrine fashion was first described in IDH‐mutant gliomas by Bunse *et al*. in 2018 [28], who also reported that the sodium transporter aids the import of R‐2‐HG. Evidence collected subsequently from Bunse *et al*. and other groups suggest that R‐2‐HG exerts its intracellular effects on T cells through metabolic rather than epigenetic reprogramming [28, 39, 40]. Probably, the exported R‐2‐HG gets imported to subclones of IDH‐mutant glioma lacking the *IDH* mutation as well, which could further explain the dominating effect of an *IDH* mutation on dictating the characteristics of IDH‐mutant gliomas. This latter aspect still needs to be examined.

### 1.5 Mechanisms of immune modulation in IDH‐mutant glioma

In addition to oncogenesis, an *IDH* mutation, through the three molecular mechanisms described above, has the potential to influence various aspects of the immune activation cycle, at two main levels of control. The first is downregulation of genes involved in immune activation in cancer cell itself, and the second is through the direct paracrine effect on TME. Downregulation of immune‐related genes is achieved either by a direct metabolic inhibitory effect of R‐2‐HG or through epigenetic reprogramming by hypermethylation of promoters of immune‐related genes. Similar to the results by Suwala *et al*., lack of response of MMR pIDH‐mutant glioma to ICB due to an immunosuppressive TME has been studied in detail [11, 28, 41, 42, 43, 44, 45, 46, 47, 48, 49, 50, 51, 52, 53].

This systematic review provides a comprehensive overview of these mechanisms through which acquiring an *IDH* mutation and subsequent generalized DNA promoter methylation, affect various aspects of immune activation cycle in IDH‐mutant gliomas. The aim of this review is to highlight this pathway as a potential actionable therapeutic target that could be considered for testing in combination with an ICB to enhance the immunological response. Such combination therapy could be promising for PMMRDIA patients, thereby, addressing the unmet therapeutic challenge of dual resistance to ICB and TMZ.

## 2 Methodology

### 2.1 Design and registration

The study design was set as a systematic review. The protocol has been registered in the International Prospective Register of Systematic Reviews (PROSPERO), under the registration ID number CRD42023461700. The review was conducted based on the Preferred Reporting Items for Systematic Reviews and Meta‐Analyses (PRISMA) guidelines [54]. By adhering to these guidelines, we aimed to enhance the clarity and completeness of our review and facilitate its reproducibility.

### 2.2 Search strategy and software used

**Fig. 2:**
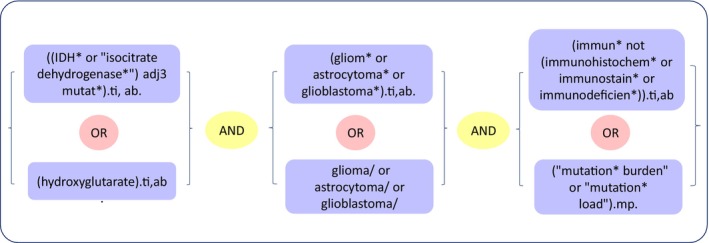
Search strategy. The figure illustrates the three search components, together with the used Boolean operators.

Medline (ovid) and Embase Databases were searched for publications published until 5th July 2023. The search strategy was based on the following three components: *IDH* mutation, tumor immunogenicity (including tumor mutational burden), and gliomas. A combination of the Boolean operators (and, or) was employed to combine the search terms effectively (Fig. 2). Two collaborators (O.A. and T.A.) carried out title and abstract screening independently to prevent screening bias, utilizing the rayyan software [55]. Subsequently, both collaborators resolved any disagreement through discussion and consensus. The full texts of the articles identified as potentially relevant, during the abstract and title screening phase, were obtained for further evaluation in the subsequent full‐text screening stage by O.A.

**Fig. 3:**
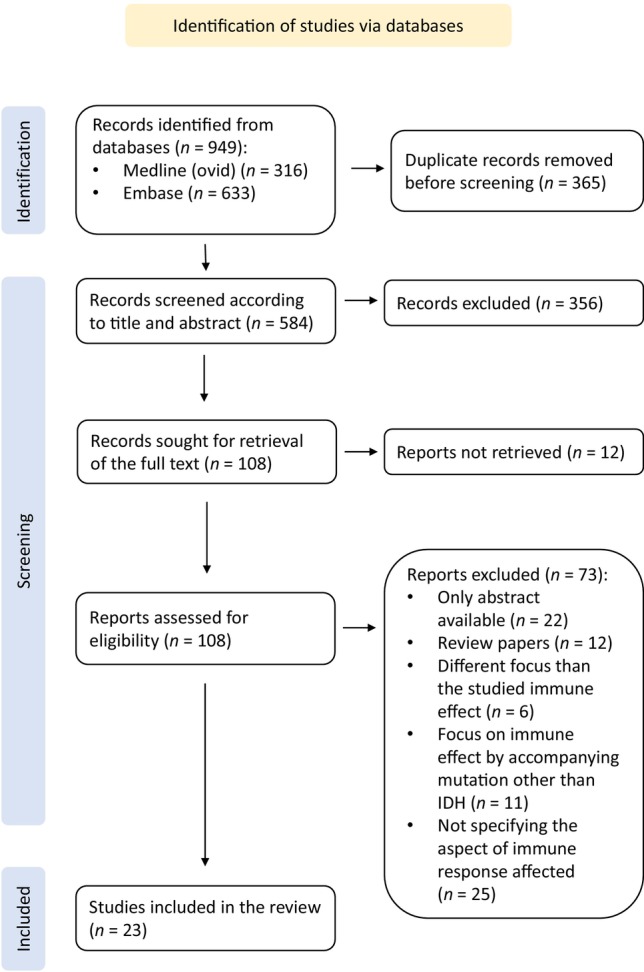
PRISMA statement. PRISMA, preferred reporting items for systematic reviews and meta‐analyses. This diagram depicts the flow of studies through the systematic review process in accordance with the PRISMA guidelines. It outlines the identification, screening, eligibility, and inclusion/exclusion of studies at each stage.

While rayyan software was used to automate the illustration of the PRISMA statement, PowerPoint was employed in creating the final version of Fig. 3, together with Fig. 2. The graphical abstract and Fig. 1 were created using inkscape software.

### 2.3 Inclusion criteria

The following inclusion criteria were established: only original research papers in English language were considered. No specific time frame for the year of publication was defined. Included papers had to be original research studies specifically investigating the impact of *IDH* mutation on the immunogenicity of glioma. To ensure the availability of comprehensive information, only papers that had the full manuscript available online were included. Only papers addressing a specific aspect of the immunity affected by *IDH* mutation were included (i.e., papers reporting an immunosuppressive TME of IDH‐mutant glioma without pointing out the specifically affected component of the immune cycle were excluded as mentioned in Section 2.4). Finally, as could be noticed from the search strategy, the systematic review included manuscripts reporting the effect of *IDH* mutation on the tumor mutational burden (TMB), even without directly addressing the effect on immunogenicity, because the effect of TMB on tumor immunogenicity is well understood.

### 2.4 Exclusion criteria

Several exclusion criteria were applied to maintain the focus and rigor of the systematic review. Firstly, studies that did not address the effect of *IDH* mutation on the immunogenicity of gliomas were excluded. For instance, studies where search terms only appeared together in the abstract but had a different primary focus were omitted. Likewise, manuscripts that specifically focused on the influence of another mutation co‐occurring in IDH‐mutant gliomas, but not *IDH* mutation itself, were also excluded from this review. Secondly, review papers were excluded from consideration, as they do not present original research findings. Thirdly, conference abstracts were also excluded as the full manuscript was not available for critical appraisal of the methods and findings. Finally, manuscripts not defining any clear aspect of the immune cycle affected by *IDH* mutation were also excluded from data extraction; however, most of them were cited in the introduction section, as they provided supporting observations that this systematic review aims to elucidate. Some studies were excluded for more than one reason.

### 2.5 Data extraction

Data extraction was conducted by a single author (O.A.), who meticulously reviewed the included studies to extract relevant information. Throughout the data extraction process, special attention was given to the molecular mechanisms of how *IDH* mutation induces the studied effect on the immunogenicity of gliomas. As downregulation of immune‐related genes within the tumor cell or affected immune cells, is the dominant mechanism of action, a special focus was placed on the role of methylation in silenced genes. In addition, considering that the aim of this systematic review is to highlight the potential therapeutic impact of targeting *IDH* mutation or methylation in combination with an ICB, manuscripts that employed an *IDH* inhibitor or a methylation inhibitor within their design were identified. Finally, critical appraisal of the methods followed in the manuscripts was assessed. This assessment aimed to determine whether the observed effects of *IDH* mutation on the TME were merely associated or if they were further validated by the investigators through *in vitro* or *in vivo* studies, demonstrating a causal relationship. Table 1 provides a summary of the studies used for data extraction, along with the aforementioned key points, and manuscripts' citations, which serve as an indicator of their significance. More details regarding the characteristics of eligible studies are provided in Section 3.1.

**Table 1:** Summary of studies included for data extraction.

	Mechanism	References	Citations up to July 2023	Downregulation of examined genes is attributed to methylation	Major limitations of the study	Studied effect modulated by a methylation inhibitor	Studied effect modulated by an IDH inhibitor
1	MHC downregulation	Lin *et al*., 2021	21	–	No validation *in vivo* or vitro	–	–
2		Louto *et al*., 2018	31	Yes	–	Yes	–
3	Lower TMB	Wang *et al*., 2020	56	Not related	Association described, without explanation	–	–
^a^		Lin *et al*., 2021	21			–	–
4		Suwala *et al*., 2021	52			–	–
5	Chemotaxis	Kohanbash *et al*., 2017	303	No	–	–	Yes
6		Amankulor *et al*., 2017	310	–	–	–	–
7		Ren *et al*., 2019	46	–	Conflicting results between two examined datasets	–	–
^a^	T cell responses	Kohanbash *et al*., 2017	303	No	–	–	Yes
8		Bunse *et al*., 2018	344	No	–	–	Yes
9		Notarangelo *et al*., 2022	41	No	–	–	–
10		Afsari *et al*., 2023	–	No	–	–	–
11	NK Cell function	Zhang *et al*., 2016	132	Yes	Validation not specifically addressing the suggested effect	Yes	–
12	Dendritic cell function	Ugele *et al*., 2019	15	–	Unclear definition of the studied effect	–	–
13		Friedrich's *et al*., 2023	14	–	–	–	–
14	Macrophage function	Gowda *et al*., 2018	31	–	–	–	–
15		Ma *et al*., 2021	13	Yes	–	–	–
16	PDL‐1 downregulation	Wang *et al*., 2016	162	–	Association described, but no validation done and no explanation provided	–	–
17		Berghoff *et al*., 2017	218	Yes	Explanation provided without validation	–	–
18		Mu *et al*., 2018	68	Yes	*In‐vitro* validation failed to maintain long lasting effect	–	–
19		Röver *et al*., 2018	60	Yes	No comparison to IDH‐wildtype	–	–
20		Kadiyala *et al*., 2021	64	–	–	–	Yes
^a^	Modulation of other immune checkpoints	Röver *et al*., 2018	60	Yes	No comparison to IDH‐wildtype	–	–
21		Liu *et al*., 2020	6	–	No validation done	–	–
22		Sørensen *et al*., 2020	4	–	No validation done	–	–
23		Zhang *et al*., 2021	4	–	–	–	–

^a^ Manuscripts appearing twice in the table.

## 3 Results

### 3.1 Eligible studies

During screening of the databases, 949 potentially relevant articles were identified. After removing duplicates, 584 articles qualified for analysis. On the basis of the available abstracts, 356 publications were rejected and 108 were accepted for comprehensive full‐text analysis. These selected full texts were then carefully scrutinized according to the pre‐defined inclusion and exclusion criteria. Eventually, 23 articles that elucidated the impact of *IDH* mutation on various aspects of immune activation were accepted for review, having met the eligibility criteria (Fig. 3). Four manuscripts addressed downregulation of antigen presentation in IDH‐mutant gliomas, either by downregulation of MHC molecules of tumor cells in two manuscripts or lower TMB of IDH‐mutant gliomas in three papers, with one of the latter being one of the formerly mentioned two manuscripts. In the realm of immune cells, three manuscripts addressed an inhibitory effect of *IDH* mutation on chemotaxis of immune cells to TME, one manuscript explored an immune escape mechanism from NK cells, two manuscripts explained the inhibition of dendritic cells, three delved into functional inhibition of T cells, in addition to the manuscript highlighting decreased T cell chemotaxis; all impacting the innate immune responses. On the other hand, two manuscripts suggested increased phagocytic activity of macrophages. Noteworthy, eight manuscripts addressed downregulation of immune checkpoint expression on tumor cells of IDH‐mutant glioma, with PDL‐1 downregulation emphasized in five of them, providing a direct mechanism for ICB resistance, in addition to the immunosuppressive TME.

### 3.2 *IDH* ‐induced effect on antigen presentation

#### 3.2.1 *IDH* ‐induced downregulation of MHC expression

In 2021, Lin *et al*. evaluated RNA‐sequencing (RNA‐seq), somatic mutation, and clinical data from 1052 low‐grade glioma (LGG) from the cancer genome atlas (TCGA) and Chinese glioma genome atlas (CGGA), and illustrated a gradual decrease in expression of several human leukocyte antigen (*HLA*) genes encoding the MHC class I proteins from the IDH‐wildtype to IDH‐mutant 1p/19q co‐deleted (current WHO nomenclature is oligodendroglioma IDH‐mutant and 1p/19q‐codeleted), with IDH‐mutant non‐co‐deleted (current WHO nomenclature is astrocytoma IDH‐mutant) occupying an intermediate position in this expression pattern. Studied genes are *HLA‐A*, *HLA‐B*, *HLA‐C*, *HLA‐DMA*, *HLA‐DMB*, *HLA‐DOA*, *HLA‐DOB*, *HLA‐DPA1*, *HLA‐DPB1*, *HLA‐DPB2*, *HLA‐DQA1*, *HLA‐DQA2*, *HLA‐DQB1*, *HLA‐DRA*, *HLA‐DRB1*, *HLA‐DRB5*, *HLA‐E*, *HLA‐F*, *HLA‐H*, *HLA‐J*, and *HLA‐L*. Methylation analysis was not performed for the included cohorts, and no validation was done to attribute the described effect to *IDH* mutation. Notably, there is a consistent pattern of differential TMB among the three described subtypes [56], as further described in paragraph 3.2.2. It could be argued that the findings of Lin *et al*. are partially supported by the findings previously published studies in 2018 by Luoto *et al*., which analyzed RNA‐seq and methylation data of 154 GBM (current nomenclature is WHO grade IV diffuse glioma) from TCGA, and reported lower expression and higher DNA methylation of *HLA‐A*, *HLA‐B*, and *HLA‐C* in IDH‐mutant GBMs (current nomenclature is WHO grade IV IDH‐mutant astrocytoma). In addition, results were validated by demonstrating an increased expression of the aforementioned genes upon inhibition of methyltransferases in BT142mut IDH‐mutant glioma cells [57].

#### 3.2.2 Lower TMB of IDH‐mutant gliomas

Lin *et al*. showed a statistically significant higher TMB in IDH‐wildtype LGG in comparison to both examined subtypes of IDH‐mutant gliomas (*P* value < 0.0001). While IDH‐mutant non‐co‐deleted group showed an intermediate value of TMB between IDH‐wildtype and the IDH‐co‐deleted mutant; no statistically significant difference was observed between TMB values of the two IDH‐mutant subtypes; namely, IDH‐mutant glioma with 1p/19q codeletion and IDH‐mutant glioma without 1p/19q codeletion [56] (current WHO nomenclatures are oligodendroglioma IDH‐mutant and 1p/19q‐codeleted, and astrocytoma IDH‐mutant, respectively). Similarly, Wang *et al*. reviewed 879 diffuse gliomas (grades II–IV) from the TCGA dataset, including 413 IDH‐mutant gliomas, and showed *IDH* mutation to be enriched in tumors with lower TMBs. It could be observed from the plot provided for tumors against their TMBs, that almost all IDH‐mutant gliomas have a TMB < 20 mutations/MB, with the vast majority having a TMB < 2 mutations/MB. No more specific conclusions could be made from this analysis, which primarily focused on the association between TMB and clinical outcome of gliomas [58]. In the analysis conducted by Swuala *et al*., as expected, PMMRDIA (*n* = 17) was reported to have higher TMB in comparison to all other subtypes of MMR‐proficient IDH‐mutant gliomas (*n* = 63). Remarkably, all analyzed PMMRDIA tumors had a TMB < 30 mutation/MB [14], which is considerably lower than what is known for TMB of other IDH‐wildtype dMMR gliomas. A comprehensive analysis of hypermutation in human cancer by the IRRDC has defined two subgroups of dMMR gliomas, with an average TMB of 380 mutation/MB and 80 mutation/MB according to the presence or absence of a secondary *POLE* mutation on top of the primary MMR mutation, respectively [59]. Despite the the association between *IDH* mutation with lower TMB illustrated by these three reports, none of the manuscripts provided an explanation or causality of lower TMB by *IDH* mutation. Indeed, *IDH*‐induced CIMP creates an abundance of mutational hotspots [60], and therefore, cannot be directly attributed to lower TMB, leaving the cause of the described association elusive. The relationship between CpG methylation and mutational rate is further elucidated in the discussion section.

### 3.3 *IDH* ‐induced inhibition of chemotaxis: quantitative reduction of infiltrating immune cells in TME of IDH‐mutant gliomas

In 2017, Amankulor *et al*. conducted a stepwise comprehensive analysis reporting downregulation of leukocyte chemotaxis in IDH‐mutant gliomas, attributing it to *IDH1* mutation. First, they started with fluorescence‐activated cell sorting (FACS) analysis (i.e., flow cytometry) of 10 IDH‐wildtype and six IDH‐mutant human glioma tissue samples, demonstrating fewer overall CD45^+^ immune cell infiltration. Further subset analysis of immune cells showed global depletion of immune infiltrates, including microglia, macrophages, dendritic cells, B cells, and T cells, compared to that of the IDH‐wildtype human gliomas. Second, differential gene expression of 91 IDH‐wildtype and 417 IDH‐mutant grade II/III gliomas from TCGA, using a threshold of two‐fold or greater and an adjusted significance *P*‐value < 0.05, identifying 1297 downregulated genes in IDH‐mutant glioma, including genes related to chemotaxis and immune migration, according to gene ontology (GO) terms [61]. Methylation analysis of identified genes was not conducted. Third, to examine if this effect is due to *IDH1* mutations, they created isogenic IDH‐wildtype and IDH‐mutant mouse glioma models whose initiating events were identical except for the expression of *IDH1* mutation. It was proved that IDH‐mutant mouse gliomas had higher levels of R‐2‐HG and DNA methylation than their wildtype counterparts. Further analysis of RNA expression data showed downregulation of some immune system processes in the IDH‐mutant mouse model, overlapping in part with identified genes from TCGA, suggesting a causal relationship between *IDH1* mutation and the identified pattern of differential gene expression. Subsequently, flow cytometry was conducted to profile the immune cells present in normal mouse brain tissue, IDH‐mutant, and IDH‐wildtype gliomas. Here, similar to human IDH‐mutant gliomas, a reduction in microglia, macrophages, monocytes, and polymorphonuclear leukocytes was observed. This was observed in addition to a negative correlation with leukocyte chemotaxis and neutrophil chemotaxis, as manifested by a Boyden chamber experiment, where cells were plated on the top of a Boyden chamber, incubated, and observed for migration, showing twice as high migration index for the IDH‐wildtype mouse gliomas. Furthermore, quantitative PCR (qPCR) showed downregulation of several cytokines; including CCL‐2, CCL‐3, CXCL‐1, CXCL‐2, CXCL‐4, CXCL‐16, GM‐CSF, IL‐1ra, IL‐2, IL‐6, IL‐16, and others. Finally, expression of these chemokines was investigated in human glioma cell lines, showing higher cytokine expression in the three patient‐derived IDH‐wildtype human glioma lines from three independent patients (U3039, U3046, and U3065) in comparison to a patient‐derived IDH‐mutant glioma cell line (TS603). This systematic approach proved that *IDH1* mutation is attributed to decreased chemotaxis in IDH‐mutant gliomas leading to decreased immune cell infiltration [62].

In 2017, another key paper by Kohanabash *et al*. described decreased chemotaxis of type 1 CD8^+^ T cells in IDH‐mutant glioma, due to downregulation of STAT1, a regulator of CXCL10, which is a chemokine of type 1 CD8^+^ T cells, that predominantly secretes interferon (IFN)‐γ [63]. They conducted a systematic multi‐step validation design comparable to the previously described study by Amankulor *et al*. [62], in addition to conducting a methylation analysis showing the described gene downregulation not to be related to epigenetic regulation [63]. An additional validation step was conducted by Kohanabash *et al*. [64], by reversing the described reductions in CXCL10 and T cell accumulation by a specific inhibitor of mutant *IDH1*.

Similarly, Ren *et al*. reported decreased expression of different immune cell‐related genes, including CD8 (*CD8A*), *CD4*, *CD19*, CD20 (*MS4A1*), CD11b (*ITGAM*), *FOXP3*, *CD163*, *CD68*, CD56 (*NCAM1*), and *S100A4* in IDH‐mutant glioma datasets of TCGA (*n* = 390) and CGGA (*n* = 312). However, their report primarily centered around the expression of the chemokine CX3CL1, which was the only chemokine with increased expression in IDH‐mutant glioma in comparison to IDH‐wildtype. Ren *et al*. suggested that CX3CL1 plays a role in promoting the recruitment of NK cells, based on its correlation with the higher levels of expression of the NK cell marker, CD56, in IDH‐mutant gliomas. Noteworthy, the statistical significance of this correlation was only observed in their analysis of the dataset from the CGGA but not TCGA [65]. Regardless of this suggested quantitative increase of NK cells in the TME of IDH‐mutant glioma, Zhang *et al*. [66] explained how *IDH* mutation allows immune escape from NK cell immunity, as explained in Section 3.4.3.

To conclude, the collective evidence indicates that TME of IDH‐mutant glioma is less infiltrated with immune cells due to IDH‐induced downregulation of chemokines, which in the case of STAT1, was shown not to be related to methylation [63]. However, the methylation effect was not examined in the other reports addressing other chemotaxis‐related genes [62, 65].

### 3.4 Dysfunction of various immune cell components

#### 3.4.1 *IDH* ‐induced suppression of anti‐tumor T cell immunity

One year after the description of reduced T cell infiltrates in IDH‐mutant glioma due to decreased chemotaxis, Bunse *et al*. described *IDH*‐induced decreased activation and proliferation of T cells. They were the first to describe a paracrine import mechanism of R‐2‐HG to T lymphocytes through sodium‐dependent SLC13A3 transporter [28]. First, they reported extracellular levels of R‐2‐HG to be fivefold higher than inside glioma cells. Later, they showed a concentration‐dependent increase in intracellular R‐2‐HG levels in human T cells, and in murine antigen‐presenting cells after exposure to R‐2‐HG. Further analyses of tumor cell lines showed a positive correlation between R‐2‐HG uptake and the expression of sodium‐dependent SLC13A3. Results were validated by reporting a 65% decrease in R‐2‐HG import upon sodium starvation, and by reporting a decrease in intracellular R‐2‐HG levels, in a concentration‐dependent manner when treated with an SLC13A3 inhibitor. Consequently, Bunse *et al*. showed reduced antigen‐specific proliferation and cytokine secretion of mouse T cell receptor (TCR) transgenic CD4^+^ T cells and TCR transgenic CD8+ T cells when exposed to R‐2‐HG *in vitro*. Next, they reported that human T cells from an IDH‐mutant glioma patient showed a concentration‐dependent reduction of *IDH1* R132H‐specific IFN‐γ production, which was also validated in a transgenic mouse model generated by them. Two types of cell cultures were produced; the first was made of activated primary mouse T cells co‐cultured with IDH‐mutant astrocytes, and the second was composed of mouse T cells co‐cultured with IDH‐wildtype astrocytes. The former showed the lower capacity to produce the effector cytokines IFN‐γ and IL‐2 after activation. Finally, investigators attributed T cell suppression to IDH‐induced alteration in calcium signaling. They observed differential downregulation of calcium‐dependent transcriptional activity of nuclear factor of activated T cells (NFAT) and NF‐κB, which are required for the transcription of IL‐2 [67], and interact with the promoter of IFN‐γ [68]. Then detected R‐2‐HG‐mediated suppression of extracellular calcium influx in stimulated CD4^+^ T cells by fura‐calcium imaging. To validate their hypothesis and assess whether the observed moderate intracellular calcium alterations are sufficient to induce the inhibitory effect on T cell proliferation, *in‐vitro* rescue experiments were conducted, restoring T cell proliferation with increasing concentrations of calcium in activated human T cells. Results were further validated in MHC‐humanized A2DR1 mice models they had previously generated [69], revealing reduced infiltration and activation of T cells, reduced IFN‐γ production, and reduced levels of NFAT. Despite that the model is generated from sarcoma cells, it supported the molecular effect of an *IDH* mutation. Noteworthy, Bunse *et al*. attributed their findings to R‐2‐HG metabolic regulation of T cells, as no epigenetic reprogramming of T cells was observed *in vitro* [28]. In addition to the R‐2‐HG induced decreased production of T cell cytokines proposed by Bunse *et al*., Notarangelo *et al*. showed R‐2‐HG to decrease degranulation of CD8^+^ T cells in a dose‐dependent manner, further impairing IFN‐γ signaling pathway. Their results were examined *in vitro* by restoring the ability of CD8^+^ T cells to degranulate upon R‐2‐HG removal at the time of T cell stimulation. In addition, they reported and validated several R‐2‐HG‐induced metabolic derangements on T cells; consistent with the described metabolic effect of *IDH* mutation on tumor cells themselves (Section 1) [39]. Recently, a more focused analysis conducted by Afsari *et al*., investigating R‐2‐HG‐induced effect on IL‐2, showed a concentration‐dependent inhibitory effect on its release, supporting the results of Notarangelo *et al*., together with suppressed NFAT‐dependent transcription, consistent with the results of Bunse *et al*. [40]. In summary, in addition to decreased T cell chemotaxis through IDH‐induced downregulation of STAT1, R‐2‐HG gets imported into the T cells through sodium‐dependent transporter, where it exerts metabolic downregulation of calcium‐dependent transcription factors decreasing IL‐2 and IFN‐γ production. Furthermore, it interferes with T cell degranulation of these cytokines, further impairing T cell responses.

#### 3.4.2 IDH‐induced immune escape from natural killer cell immunity

In 2016, Zhang *et al*. [66] conducted a multistep validation design study, suggesting that IDH‐mutant gliomas escape NK cell immune surveillance by downregulating NK group 2D ligand (NKG2DL). NKG2DL are membrane‐bound proteins expressed on tumor cell surfaces that are recognized by NKG2D receptors on NK cells and CD8^+^ T cells, mediating cytotoxic immune recognition, independent of tumor‐specific antigens. Zhang *et al*. first observed downregulation of NKG2DL by analyzing RNA‐seq data from 286 diffuse glioma patient samples from TCGA, which was also correlated with promoter hypermethylation. Second, they validated their results by examining the differential expression level of NKG2DL in IDH‐mutant and wildtype glioma cell lines, with the former exhibiting > 5 times lower expression (*P* value < 0.001). Thirdly, they validated their conclusion by performing mixed allogeneic co‐cultures of donor NK cells and IDH‐mutant/wildtype astrocytes, and imaged astrocytes using phase‐contrast micrography after 72 h of co‐culture, showing 97% of the IDH‐mutant astrocytes to be viable and adherent, compared to 54% of wildtype astrocytes (*P* value = 0.01). As they attributed their results to DNA hypermethylation, they concluded their study by applying decitabine‐mediated hypomethylation to restore expression in IDH‐mutant glioma cells, suggesting its clinical potential to sensitize IDH‐mutant gliomas to NK cell‐mediated immune surveillance in patients with IDH‐mutant gliomas [66]. Hence, the focus of Zhang *et al*. was not to assess the direct effect of R‐2‐HG on NK cells, but rather their impaired binding to tumor cells, which was attributed to ligand impairment on the tumor sites and not NK cells. None of the identified studies examined if R‐2‐HG was imported into NK, or studied its direct effect on NK cells.

#### 3.4.3 R‐2‐HG limits the activation and antigen presentation by dendritic cells

Similar to T‐cells, dendritic cells (DCs) are capable of uptaking R‐2‐HG, as shown by Ugele *et al*. in 2019 [70], which reported a 100‐fold increase in the intracellular levels of R‐2‐HG in DCs under treatment with 10 mm R‐2‐HG. However, mechanistic insights of this intracellular uptake remained elusive. Subsequently, they showed downregulation in IL‐12 levels owing to the intracellular accumulation of R‐2‐HG. Notably, IL‐12 plays a crucial role in T cell activation by improving the type 1 T helper cell response, promoting the expansion and survival of activated T cells and NK cells, enhancing CD8+ T cell homeostatic expansion, leading to enhanced TCR‐induced signaling and cytokine production, and increasing CD8^+^ T cell cytolysis, survival, and proliferation [71, 72]. Ugele *et al*. conducted their analysis on leukapheresis‐isolated peripheral blood mononuclear cells of consented healthy donors from the University Hospital Regensburg. They first showed decreased mRNA expression of both *IL‐12A* and *IL‐12B* genes post‐R‐2‐HG treatment in a dose‐dependent manner. While expression levels of *IL‐12A* and *IL‐12B* genes were significantly decreased 4 h after the treatment, the effect returned to baseline level in 24 h for *IL‐12A*, but persisted for *IL‐12B*. While the report described reduced secretion of IL‐12 from DCs after R‐2‐HG treatment, the underlying cause for reduction in IL‐12 secretion remains unclear. While no methylation analysis was conducted in this study, investigators attributed their findings to the metabolic effect of R‐2‐HG and validated this based on the fact that lipopolysaccharide (LPS) is a potent stimulant of DCs [73]. Considering that, Ugele *et al*. showed that R‐2‐HG reverses the effect of LPS on cellular respiration which promotes DC stimulation. Finally, they showed that the addition of ATP synthase inhibitor, oligomycin, to DC cultures to increase IL‐12 secretion, partially reverted the effect of R‐2‐HG. This suggested that R‐2‐HG‐induced changes in cellular respiration may contribute to the diminished IL‐12 secretion [70]. In 2022, Friedrich *et al*. suggested that the biological function of DCs in glioma is not restricted to priming peripheral T cells, but rather blood‐borne monocytes accumulate intratumorally during glioma progression and experience a glioma genotype‐dependent DC education. Altogether, this might result in diverging immunological capacities, which is not the scope of this systematic review [74].

#### 3.4.4 IDH‐induced effects on macrophages

In 2018, Gowda *et al*. described decreased CD47 expression and its role in IDH‐mutant glioma, by examining mRNA expression *in vitro* and *in silico* utilizing the TCGA dataset. Given that CD47 is a transmembrane glycoprotein in a tumor cell that delivers an inhibitory signal for macrophage phagocytosis, they subsequently examined microglia's capability to engulf IDH‐mutant cells with reduced CD47 levels. As expected, microglia exhibited a stronger phagocytic response toward the U87MG cells overexpressing mutant *IDH1* compared to cells expressing wildtype *IDH1* [75]. No methylation analysis was conducted in this study. Notably, tumor‐associated microglia/macrophages (TAMs) are the main innate immune effector cells in malignant gliomas, and they have both pro‐ and anti‐tumor functions, with their plasticity being partially dictated by underlying oncogenic mutations [76]. This finding of Gowda *et al*. was further supported by Ma *et al*. [76], which demonstrated for the first time that glioma cells carrying heterozygous *IDH1* R132H mutation switch TAMs toward a phagocytic anti‐tumor phenotype by downregulating ICAM1/CD54 pathway. Similar to Gowda *et al*., Ma *et al*. also validated their findings *in vitro* and *in silico* utilizing the TCGA dataset. Similar to CD47, ICAM1 is expressed on the tumor cells and not the macrophages. Ma *et al*. [76] additionally examined the methylation data and attributed *ICAM1* downregulation to hypermethylation of its promoter. In conclusion, both studies suggest enhanced anti‐tumor phagocytic activity of microglia in IDH‐mutant glioma in comparison to IDH‐wildtype, which contributed to better prognosis of the former. Noteworthy, both studies addressed the modulation of tumor cell's ligands interacting with microglia, but did not examine additional direct effects of R‐2‐HG on TAMs themselves, which might provide more insights into the immune function of TAMs in the TME of IDH‐mutant gliomas.

### 3.5 Downregulation of immune checkpoints

#### 3.5.1 Epigenetic downregulation of PDL‐1

Downregulation of PDL‐1 in IDH‐mutant glioma was first described in 2016 by Wang *et al*., which analyzed transcriptomic data of 976 glioma samples of grades II–IV, including 301 microarray data from the CGGA project and 675 RNA‐seq data from TCGA. Their analyses showed that IDH‐mutant glioma has lower expression of PD‐L1 (*CD274*) in comparison to IDH‐wildtype, across all grades, with significant adjusted *P*‐value of < 0.05 for grade IV (both CGGA and TCGA), and grade III (TCGA). No methylation data were analyzed in this study [77]. Similarly, Berghoff *et al*. reported statistically significant (*P* < 0.001) higher PD‐L1 expression among all grades (grades II–IV) of Vienna glioma cohort [*n* = 174, composed of 43 WHO grade II/III gliomas and 131 GBM (current nomenclature is WHO grade IV diffuse glioma)]. In addition, they examined a subset of glioma from the TCGA database for which the methylation data together with RNA‐seq data (*N* = 51 IDH‐wildtype and 4 IDH‐mutant) was available. Resultant analyses showed a reduced PD‐L1 gene expression associated with increased promoter methylation (Spearman correlation coefficient − 0.36; *P* < 0.01) in the LGG cohort [45]. In 2018, Mu *et al*. concluded the same results by examining tumors from 35 adult patients with WHO grade II/III glioma together with 15 primary GBMs (current nomenclature is WHO grade IV diffuse glioma) from three Chinese hospitals. Furthermore, IDH‐wildtype tumors showed relatively higher levels of PD‐L1 gene and protein levels in both primary LGGs and GBMs (current nomenclature is WHO grade IV diffuse glioma) as compared to IDH‐mutant tumors. While their analyses did not provide specific *P* values, they identified two CpGs within the PD‐L1 promoter (cg15837913 and cg19724470) that exhibited differential methylation patterns between normal brains and tumors as well as IDH‐mutant and wildtype gliomas. Subsequently, they validated their findings *in vitro*, by adding R‐2‐HG (0, 3, or 6 mm) daily to the cell culture of an IDH‐wildtype GBM line U87. They observed a transient increase in DNA methylation of both CpG sites 24 h after R‐2‐HG was added. However, DNA methylation was reversed at 48 h, in addition to a surge in DNA methylation beta‐value of the non‐treated cells at the same time point. Investigators could not provide an explanation for the results observed at 48 h, as mechanisms underlying the dynamic methylation patterns that dictate spatial and temporal gene expression are still not completely understood [78]. In 2021, Kadiyala *et al*. supported the previous findings by showing an increased PD‐L1 expression in response to R‐2‐HG inhibition, up to similar levels as observed in IDH‐wildtype gliomas. Interestingly, they already combined R‐2‐HG inhibition with radiotherapy, TMZ, and anti‐PDL1 ICB, and observed complete tumor regression in 60% of IDH‐mutant glioma‐bearing mice. Based on their results, they propose utilization of IDH inhibitor and ICB in the clinical practices to treat IDH‐mutant glioma [79], which indeed would be particularly valuable for the PMMRDIA group, which is otherwise resistant to TMZ. Noteworthy, Suwala *et al*. [14] also reported PMMRDIA tumors to have scarce or no expression of PD‐L1 in a small percentage of tumor cells. Despite this, the molecular pathways and TME of this particular group of IDH‐mutant glioma are not yet known to us.

#### 3.5.2 Downregulation of other immune checkpoints

In 2018, Röver *et al*. expanded RNA expression analysis and DNA promoter methylation analysis to more ICB‐related genes including PD‐L1 (*CD274*), PD‐1 (*PDCD1*), PD‐L2 (*PDCD1LG2*), and CTLA‐4 (*CTLA4*), utilizing data from TCGA, focusing on subgroups of IDH‐mutant LGGs (*n* = 419). They first showed a statistically significant inverse correlation of mRNA expression levels with promoter methylation for the three genes related to PD‐L1, PD‐L2, and CTLA‐4. Subsequently, they analyzed the difference in methylation among three methylation classes of LGGs: LGm1, LGm2, and LGm3 (according to classification by Ceccarelli *et al*. [80]); while exact corresponding WHO classes are not clear from Ceccarelli's paper, it could be understood that LGm3 is 1p/19q co‐deleted, and both LGm1 and LGm2 are non‐co‐deleted, with enrichment of pediatric cases among LGm1 group [80]. Among the three studied groups, promoter methylation of all PDL‐1, PDL‐2, PD‐1, and CTLA‐4 was the lowest in LGm1. In addition, they correlated methylation patterns of examined checkpoints to various clinical and molecular characteristics of gliomas, where PD‐1 methylation qualified as a strong prognostic factor together with age [81]. The latter finding was supported by Liu *et al*. in 2020, who showed upregulation of PD‐1 expression in IDH‐mutant GBM (current nomenclature is WHO grade IV IDH‐mutant astrocytoma) and IDH‐wildtype GBM, based on their analysis of PD‐1 transcriptional expression data of 1323 glioma from the CGGA and TCGA datasets and a local hospital cohort. No methylation analysis was conducted, and no validation was done, as the focus of the analysis was on the correlation between expression pattern and clinical behavior [82]. Another protein of the B7 family of checkpoints, B7H3, has the highest expression compared to other members of the B7 family in GBM (current nomenclature is WHO grade IV diffuse glioma) as shown by Zhang *et al*. [83]. In a subsequent study, the same group examined its differential expression according to *IDH* status, and showed statistically significant lower mRNA expression in IDH‐mutant LGG of TCGA cohort in comparison to IDH‐wildtype, together with lower expression in IDH‐mutant GBM (current nomenclature is WHO grade IV IDH‐mutant astrocytoma) of TCGA, without statistical significance due to small number. Additionally, they showed lower protein expression in fresh tumor samples, as well as glioma cell line U87 treated with cell‐permeable 2‐HG [84]. Consistent with its previously identified involvement in angiogenesis in pancreatic and colorectal carcinoma [85, 86], Zhang *et al*. [84] also showed that B7H3 expression correlates with VEGFA and MMP2 expression, which are all downregulated in IDH‐mutant LGG, based on the mRNA data analysis of the TCGA dataset and reduced protein levels in *IDH1* R132H U87 cells compared to its wildtype counterparts. Furthermore, given the galectin‐9 (Gal‐9)/T cell immunoglobulin and mucin‐domain containing‐3 (TIM‐3) pathway is gaining significant attention in cancer immunotherapy as an additional inhibitory checkpoint system, its expression in IDH‐mutant glioma was also examined by Sørensen *et al*. in 2020. It is understood that TIM‐3 is present in various T cell subsets and plays a crucial role in promoting T cell tolerance through its interactions with ligands, including Gal‐9. In addition, TIM‐3 has been associated with T cell exhaustion and dysfunction, particularly when expressed alongside PD‐1. In the analysis conducted by Sørensen *et al*. on a local cohort of grades III/IV glioma (36 IDH‐mutant and 36 IDH‐wildtype) together with a validation TCGA cohort, *IDH* mutation was significantly linked to lower levels of TIM‐3^+^ cells and decreased interactions between TIM‐3^+^ T cells and galectin‐9^+^ microglia/macrophages, as shown by lower TIM‐3 mRNA expression and IHC staining. No methylation analysis was conducted, and no validation was conducted *in vitro* or *in vivo* to show a direct effect of *IDH* mutation on the expression of TIM‐3 [87].

## 4 Discussion

IDH‐mutant gliomas are characterized by an immunosuppressive TME. By conducting a systematic review summarizing available evidence of *IDH*‐induced effects on the immunogenicity of IDH‐mutant glioma, three main mechanisms were identified; namely, (a) epigenetic, (b) metabolic, and (c) paracrine. Extracted evidence from the 23 studies included in this systematic review are classified into nine categories. The first two categories are related to decreased antigen presentation either by decreased MHC expression, or lower TMB. In addition to the described methylation‐induced downregulation of MHC class I molecules by Luoto *et al*., it could be argued that G‐CIMP might promote a more generalized gene silencing of various cancer‐associated neoantigens, similar to the hypermethylation of CpG islands located at the promoters of melanoma‐associated antigens (MAGE) and other cancer testis antigens (CTA), presenting a common mechanism of their downregulation in various solid tumors and hematologic malignancies [88, 89]. To the best of our knowledge, this effect has not been studied in IDH‐mutant glioma, despite their characteristic G‐CIMP phenotype.

While our results show an association between *IDH* mutation and lower TMB in comparison to IDH‐wildtype gliomas of comparable MMR status, the underlying mechanisms responsible for this association remain unclear. It is understood that IDH‐induced G‐CIMP have abundant mutational hotspots which cannot be attributed to lower TMB of IDH‐mutant gliomas. 5‐Methylcytosine (5‐mC) is the most common DNA modification found in the eukaryotic genome, and it is estimated that ~ 90% of 5‐mC occur within the CpG dinucleotides in vertebrate genomes [60]. Likewise, it is thought that the majority of Cs within CpGs are methylated [60]. While C is the most unstable nucleobase, 5‐mC is considered even more prone to spontaneous deamination [90, 91, 92], producing 5‐mC>T mutations, which predominate the spectra of spontaneous base substitutions in mammalian cells as well as in *Escherichia coli* [93]. In fact, unmethylated C deaminates to Uracil (U), while 5‐mC deaminates to T [4]. The latter event results in difficult‐to‐repair mismatches [93], which further explains why CpGs are considered mutational hot spots, harboring around 35% of all mutational events of the genome [60]. In addition to spontaneous cytosine deamination, cells additionally incur a large number of G:C>A:T transitions upon DNA replication, throughout the whole genome (not concentrated in CpGs). These become particularly abundant in the case of deficient MMR machinery, where they are estimated to occur at a significantly higher rate than spontaneous deamination of 5‐mC which concentrates at CpG dinucleotides [94]. This is why hypermutation is defined not only by the abundance of mutations but also by a strong signature of G:C>A:T transitions at CpG and non‐CpG sites [12]. Indeed, the three identified studies addressing lower TMB in IDH‐mutant gliomas suggest that there must be a mechanism other than G‐CIMP affecting the mutational rate in IDH‐mutant glioma. The presumed mechanism might be decreasing the rate of replication errors in non‐CpG sites. Indeed, there are several other characteristic genetic and epigenetic mechanisms in IDH‐mutant gliomas that could affect the rate of DNA replication, such as histone methylation and *ATRX*‐induced chromatin modulation, whose effect on TMB of IDH‐mutant glioma is not examined in this systematic review addressing the effect of *IDH* mutation itself.

Thirdly, decreased chemotaxis of various types of immune cells was well elaborated by the two milestone studies of Amankulor *et al*. and Kohanabash *et al*. Ren *et al*. also supported the findings of the previous two groups on a generalized decrease in chemotaxis and suggested an increased chemotaxis of NK cells to IDH‐mutant gliomas. Regardless of this, Zhang *et al*. explained how *IDH* mutation allows immune escape from NK cell immunity by downregulating NKG2DL, which is the fourth aspect identified by our systematic review. NKG2DL expression is stress‐induced and is known to occur following oncogenic transformation regardless of specific tissue type [95]. It is not surprising that its downregulation has been described as an immune escape mechanism in other cancers as well [96, 97, 98]. Similar to IDH‐mutant glioma, its downregulation by methylation has also been described in AML [98]. Fifth, decreased interaction with immune cells in the two papers addressing downregulation of CD47 and ICAM1 results in an increased phagocytic activity of TAMs. Indeed, the expression of CD47 is a general mechanism through which human solid tumor cells evade phagocytosis [99]. Similarly, ICAM1 is a member of the immunoglobulin supergene family that interacts with β2 integrins, and plays a critical role in suppressing immune activation and inflammation [100]. Against a vast majority of evidence consistent with immune suppressive effect of *IDH* mutation, Gowda *et al*. and Ma *et al*. identified that the anti‐tumor phagocytic activity of microglia could be attributed to a better prognosis of IDH‐mutant gliomas in comparison to IDH‐wildtype.

Sixth and seventh, in contrast to studies addressing NK and TAM immunity only from the angle of their interaction with tumor ligands, import of R‐2‐HG to T cells and dendritic cells has been well studied, where it has been found to exert significant metabolic and functional derangements contributing to immunosuppressive effect. Indeed, elevated serum levels of R‐2‐HG have been reported in several types of IDH‐mutant tumors [25, 26, 27], explaining how the net outcome of R‐2‐HG accumulation results in survival benefit of tumor cells, despite its potential to disturb several metabolic pathways, thereby, diminishing cellular fitness. It would not be surprising if R‐2‐HG was similarly identified to be imported into NK cells and TAMs, where it could further affect their immunological function. In addition, R‐2‐HG might also be imported into tumor cells of the subclones lacking *IDH* mutation, within IDH‐mutant gliomas, exerting its epigenetic and metabolic effect there as well. Altogether, this, in part, explains how an *IDH* event in a glioma behaves in a predominant manner to exert distinguished molecular and metabolic phenotypes.

Eighth and most interestingly, the downregulation of PDL‐1 within IDH‐mutant gliomas, which could be directly responsible for the resistance of IDH‐mutant gliomas to Nivolumab, including PMMRDIA. Based on the literature, not enough evidence was found to validate the causal effect of *IDH* mutation in downregulating PDL‐1 expression. While this is not a counterargument, *in vitro* or *in vivo* validation along with testing the efficacy of proposed combination therapy of a DNA methylation inhibitor or an IDH inhibitor in combination with ICB is warranted. Lastly, other immune checkpoints were found to be downregulated in IDH‐mutant gliomas, due to hypermethylation, with some variation in the degree of downregulation between different subclasses. This suggests that astrocytoma has a more prominent downregulatory effect of the various studied immune checkpoints shown by Röver *et al*., in comparison to 1p/19q co‐deleted oligodendroglioma, which is particularly pertinent in PMMRDIA. Taken together, these findings will guide the use of various ICB‐based treatment options.

## 5 Conclusions and perspective

In conclusion, the downregulatory effect exerted on immune‐related genes of IDH‐mutant glioma cells is attributed to hypermethylation; including downregulation of MHC class 1 molecules, NKG2DL interaction with NK cells, interaction of CD47 and ICAM1 with TAMs, together with downregulation of immune checkpoints such as PDL‐1 and PDL‐2. This is not surprising given the characteristic G‐CIMP, which might additionally affect the expression of other tumor antigens. While paracrine effect of R‐2‐HG on immune cells was primarily metabolic rather than epigenetic, the downregulation of PD‐1 and CTLA4, which are expressed on immune cells, can be attributed to hypermethylation, suggesting that R‐2‐HG could also lead to epigenetic reprogramming in a paracrine manner, as summarized in Table 1. In the last decade, precision therapeutics targeting *IDH* mutation and DNA methylation have been employed as differentiation agents in cancer treatment, and have been approved by the Food and Drug Administration (FDA) for the treatment of myelodysplastic syndrome (MDS) and AML [101, 102]. In IDH‐mutant gliomas, both modalities have shown promising results in preclinical and clinical trials [103, 104]. It could be argued that while epigenetic modulators may effectively counteract the hypermethylation phenotype associated with an *IDH* mutation, their impact on other cancer‐related features in IDH‐mutant tumors, such as metabolic reprogramming and DNA repair pathways, remains uncertain [11].

In addition to their anti‐tumorigenic effect, it can be deduced that both *IDH* and methylation inhibitors aid in the management of IDH‐mutant glioma by additionally modulating its TME, as summarized in Table 1. In the contemporary landscape of advancing combinatorial therapeutic approaches with ICB to enhance efficacy and tackle resistance, the potential combination of *IDH* inhibitors and/or methylation inhibitors with ICB emerges as a promising strategy for IDH‐mutant gliomas generally, and specifically for PMMRDIA; where it holds particular promise, addressing the dual resistance of PMMRDIA to ICB and the standard‐of‐care chemotherapeutic agent TMZ. Indeed, the efficacy of methylation inhibitors in combination with ICB has been already examined in several other malignancies [105, 106, 107, 108]. While our review focused on the potential of using an *IDH* inhibitor and/or a methylation inhibitor in combination with an ICB, there are other IDH‐targeted therapeutics including the *IDH1* R132H specific vaccine [4, 109], which could be used as alternative therapy options.

However, there are two major limitations of this systematic review. First, we aimed at collecting comprehensive evidence regarding the effect of *IDH* mutation on the immunogenicity of glioma, and therefore, excluded manuscripts that did not directly address the effect of *IDH* mutation. While this strategy helped to maintain the focus of our review, it would be useful to comprehend our results together with the collective available evidence regarding the immune effect of other known driver mutations accompanying *IDH* mutation in glioma, such as *ATRX*. Based on our PRISMA statement, (Fig. 3) eleven manuscripts were excluded from this review for this reason. Secondly, while we aim at exploiting the extracted evidence, to suggest a preclinical trial of a combinational regimen composed of an *IDH* inhibitor and/or methylation inhibitor together with an ICB for PMMRDIA, it needs to be taken into consideration that PMMRDIA has a distinct methylation profile that clusters separately from other groups of IDH‐mutant glioma, and therefore some of the identified findings regarding methylation‐induced downregulated genes might not hold true for PMMRDIA; thus, these need to be tested in methylation data of the PMMRDIA cohort. However, the majority of our findings were already validated *in vitro* and/or *in vivo* for their causal relationships with an *IDH* mutation, and therefore are likely to be true for PMMRDIA too.

On the other hand, this is the first systematic review summarizing the effect of *IDH* mutation on the immunogenicity of IDH‐mutant gliomas. It is also the first manuscript providing insights regarding the unmet therapeutic challenge of PMMRDIA, suggesting a preclinical trial to evaluate the effectiveness of a combination therapy involving an ICB in conjunction with an *IDH* inhibitor or DNA methylation inhibitor for PMMRDIA.

## Conflict of interest

None.

## Author contributions

O.A. conceptualized the work, carried out the title and abstract screening together with full‐text screening, extracted data, drafted the manuscript, and created the illustrations. T.A. carried out title and abstract screening and reviewed the manuscript. S.M.P. reviewed the manuscript. All authors have reviewed and agreed on the final version of the manuscript.
